# Improved model for intrusion detection in the Internet of Things

**DOI:** 10.1038/s41598-025-92852-6

**Published:** 2025-07-01

**Authors:** Marina S. Amine, Fayza A. Nada, Khalid M. Hosny

**Affiliations:** 1 Department of Information Technology, Faculty of Computers and Information, Suez University, Suez, 8151650 Egypt; 2 Department of Computer Science, Faculty of Computers and Information, Zagazig University, Zagazig, 44519 Egypt

**Keywords:** IoT, Intrusion detection, Deep learning, Data augmentation, Engineering, Mathematics and computing

## Abstract

The Internet of Things (IoT) includes many devices generating vast amounts of data that need extensive computation. IoT has several definitions, but the most popular refers to multiple devices, objects, and sensors all connecting via a network to exchange data. IoT has become more efficient in processing large amounts of data in less time than before because it does not require human intervention. Recently, IoT technologies have improved intelligent systems, such as smart cities, healthcare, smart homes, and more. Unfortunately, IoT faces several security issues and is vulnerable to attacks. To prevent damage or losses, we must detect such anomalies. Internet of Things (IoT) devices are developed daily, leading to increased security vulnerabilities. This work presents an improved deep learning (DL) model for intrusion detection in Internet of Things (IoT) environments to improve accuracy and generalization. It uses convolutional neural network (CNN) capabilities to achieve that. The proposed model was tested on several benchmark datasets and demonstrated notable advances over alternative DL as Long-Short Term Memory (LSTM) and machine learning techniques like Decision Tree (DT). The proposed CNN-based model integrates data augmentation and regularization to prevent overfitting. Furthermore, the model achieves a high precision rate equal to 1, and the average precision to multi-class reaches 82%, which is essential to reduce false positives in real-world applications. This work sets a new standard for future IDS development research and emphasizes how deep learning can be used to improve IoT security. Our enhanced model offers an efficient and scalable way for detecting over 10 attacks to defend IoT networks against constantly changing cyber threats by addressing IoT environments’ particular difficulties.

## Introduction

The Internet of Things (IoT) has recently been included in everything we use daily, such as our homes, cars, and other objects. IoT has various features, services, and applications that changed people’s lives and shaped the world. Smart systems, such as those in homes, cities, and other areas, have improved with the development of IoT technologies^1^. IoT is vulnerable to attacks and has several security issues. Denial of Service attacks (DoS) and Distributed Denial of Service (DDOS) are common security attacks that make a resource, network, or device unavailable to authorized users. Each layer in IoT is susceptible to threats and attacks, which can be passive or active attacks. New security challenges appeared because of the wide distribution of IoT systems. Privacy and security are challenges in IoT because of the vast data it collects; one of the significant solutions for these challenges is using machine learning and deep learning methods, which learn from the results of trained data to increase decision-making performance. Also, trained data can differentiate between regular benign or malicious traffic. This differentiation enables the detection of abnormal behavior to prevent unauthorized access. IoT has a large amount of data, which makes it difficult to improve security and meet all requirements such as performance, reliability, and cost-effectiveness. If one of the features is improved, this will affect others, increase security checks and protocols, increase latency, and increase the number of connected devices, increasing the number of attackers who gain access.

This paper considers this challenge when choosing features and dealing with several attacks. We didn’t select one type of attack or a small group of features to spot and improve regardless of the other attacks and features. However, we carefully improved the model that deals with several different attacks while considering how to improve the performance of all other requirements. Most devices in the market now don’t have security features like firewalls, anti-virus, etc., so, in IoT security, an intrusion detection system (IDS) is the best solution for detecting those attacks.

An IDS is software that runs on IoT devices. It detects and monitors any potentially harmful activity on a device and manages how the network’s devices behave. When an intrusion (attack) is detected, the IDS alerts the device administrator, who isolates the malicious devices to prevent further intrusions. IDSs help ensure device security in an IoT environment^2^. Here, the compute unit stores traffic flow in its database for future studies or model re-calibration, and the central unit classifies message integrity.

IDS can be categorized depending on the type of intrusions found. They are divided into four categories: signature, anomaly, specification, and hybrid approach-based intrusion detection systems. However, there are other design choices for IDS for IoT systems, including those based on the data source, environment, architecture, and detection mechanism^3^. In recent years, IDSs have used ML\DL methods; those techniques are instrumental in detecting known attacks and predicting unknown attacks. Quality of services (quality of service) and security levels of the IoT network can be improved intelligently. A complete overview of anomaly detection for IoT will be given in this paper, including an analysis of various techniques, methodologies, and challenges associated with detecting anomalies in an IOT environment. Different ML&DL models have been used to find the best model for anomaly detection^3,4^. Comparing them to get the model with the highest accuracy to detect and prevent malware attacks in resource-constrained IoT devices. Multiple models have been chosen to meet users’ needs. The structure is built as follows: section “Related work” summarizes the several techniques used by IDS and highlights their key difference while also providing some light on previous related work. The suggested model, findings, and analysis of earlier research are presented in section “Proposed model”. Section “Conclusion and future works” is for conclusion and future challenges; finally, the References are in Sect. 5.

## Related work

**Fig. 1:**
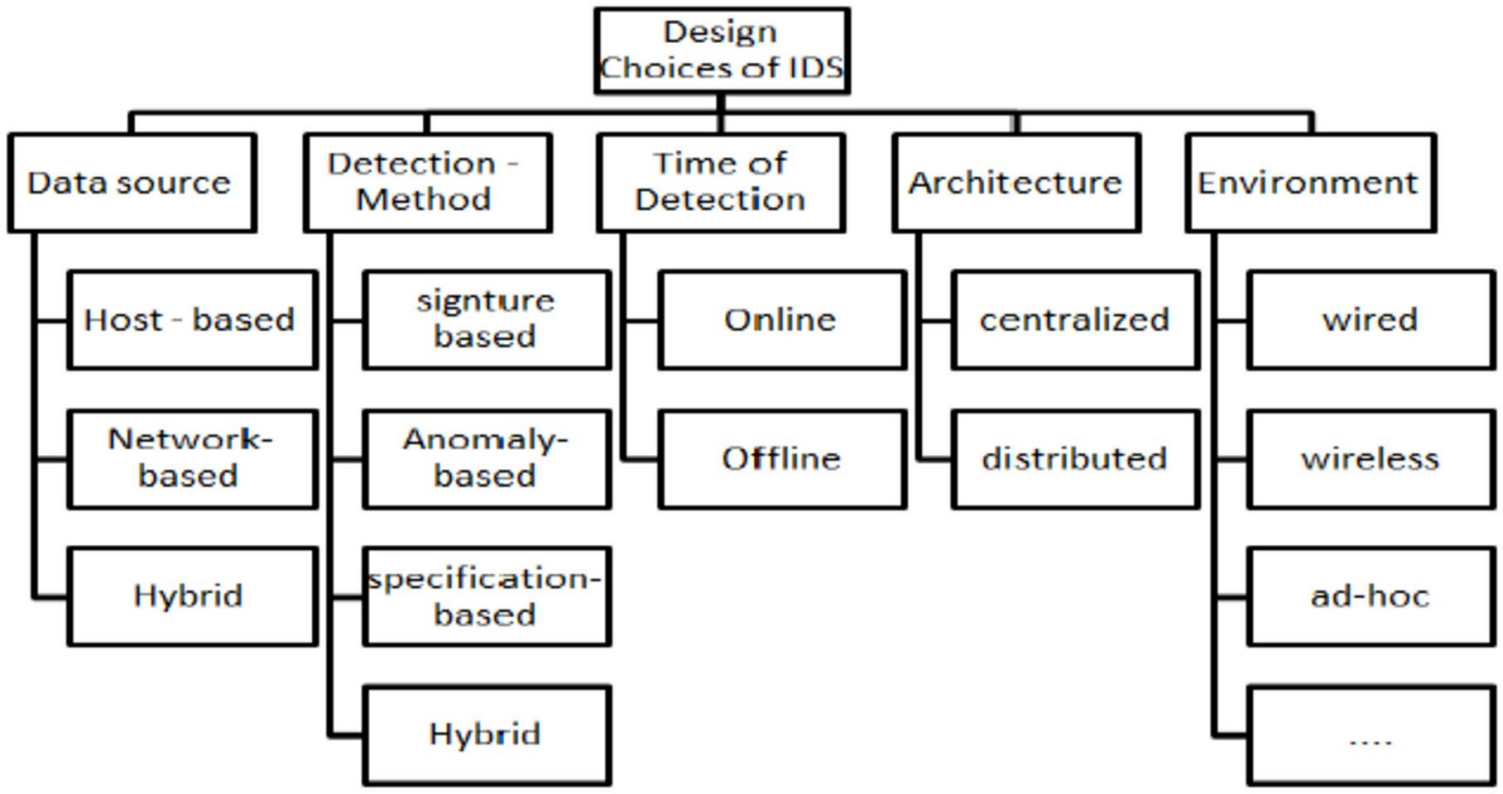
Design Choices IDSs for IOT.

IDSs for IoT are arranged based on different categories^2^. Some strategies are based on placement and detection methods^5^. Most previous related work discussed finding the best design choice IDS for IoT. Based on the detection method, data source, time of detection, architecture, and environment, Fig. 1 shows most design choices. According to data sources, the first classification is host, network, or hybrid. Signature, anomaly, specification, or hybrid are subcategories in detection methods. The detection time can be online or offline, while the Architecture can be centralized or distributed. The environment can be wired, wireless, or ad hoc.

According to Fig. 1, every IDS design choice has its type, like IDS for IoT, based on the detection method discussed^3^.

### Detection methods of IDS

There are different ways to classify IDS detection methods, as shown in Fig. 1. IDSs are classified into four types^4^, which will be explained in this section.

#### Detection based on signature

Signature-based detection methods include repository attacks, which compare system actions or network data. An alarm is raised when a detected attack matches the same one in the repository attacks. This approach can be helpful for predefined attacks in which signatures are accessible in the repository but cannot detect (zero-day) new attacks^6–8^.

Certain studies, such as^9^, suggested using an artificial immune system (AIS) to compensate for signature-based techniques’ shortcomings. This technique used immune cells to develop detectors that rely on attack signatures or patterns to determine if a packet is harmful by classifying its elements as self or non-self. Because of constant system monitoring, the system can take in new patterns. However, it’s doubtful whether such a detection method might work in an IoT context with limited resources.

In^10^, the authors addressed the resource constraint issue with intrusion detection systems based on signature using a different Linux machine that ran a customized Suricata-based intrusion detection system. However, the authors offered no hints about how to keep it updated. In^11^, they suggested changes to signature matching methods, building on the work published in^10^.

#### Detection based on anomaly

System architectures for anomaly-based detection in the monitored environment are built on a baseline of normal behavior^8,11^. The system’s current behaviors are then compared to this usual baseline. When an abnormality exceeds the permitted threshold, an alarm is set and recorded but not classified.

Detection methods based on signatures are not as successful as anomaly-based methods in identifying new attacks. One limitation of this technique is the difficulty in creating the baseline profile of normal behavior, which results in higher false favorable rates^12–14^. Anomaly-based detection solutions use machine learning (ML) algorithms to give a standard baseline profile of the systems they monitor. Using ML algorithms in IoT scenarios with limited energy and resources is still difficult because of the large amount of processing power needed to train and test them.

#### Detection based on specification

Detection methods according to specification and anomaly are based on the idea that out-of-range deviations are identified by deploying a technique to describe a system’s typical behavior and comparing it to the system’s current activities. However, with methods based on specification, human experts determine normal behavior manually by using a repository of relevant ranges of deviations and regulations. In contrast, techniques based on anomalies discover normal behavior through machine learning^15^. The false positive rate here is decreased than in detection based on anomaly methods^14^. In addition, after defining a rule set, these strategies do not require a learning phase^15^; they are not sensitive to setting changes. They may have specification errors^16^.

#### Hybrid detection

According to the previously discussed strategies, the hybrid-based detection methods reduce the disadvantages and increase the benefits of identifying new and current assaults^17^. This IDS was created using these two approaches to balance processing and storage needs for anomaly and signature-based detection algorithms. They compare the computing costs of approaches based on anomalies and the detection approaches based on the signature’s storage costs.

### Machine learning (ML) and deep learning (DL) techniques

#### ML techniques for IDS

**Fig. 2:**
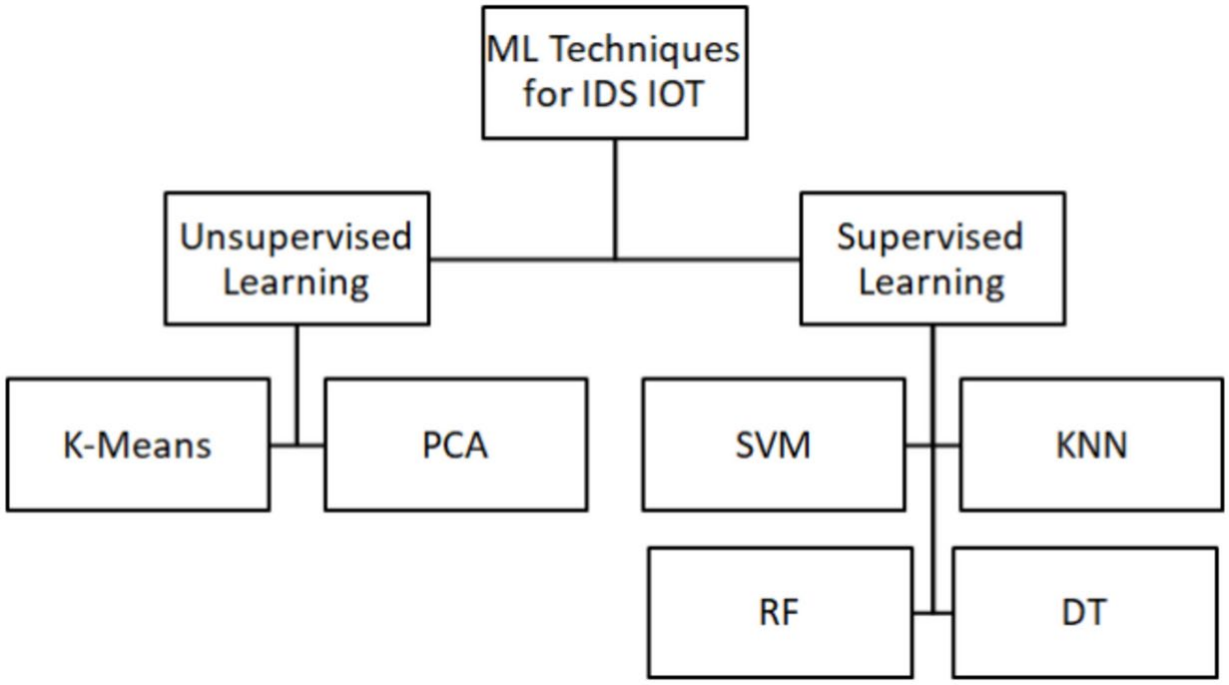
ML techniques of IDS for IOT.

**Fig. 3:**
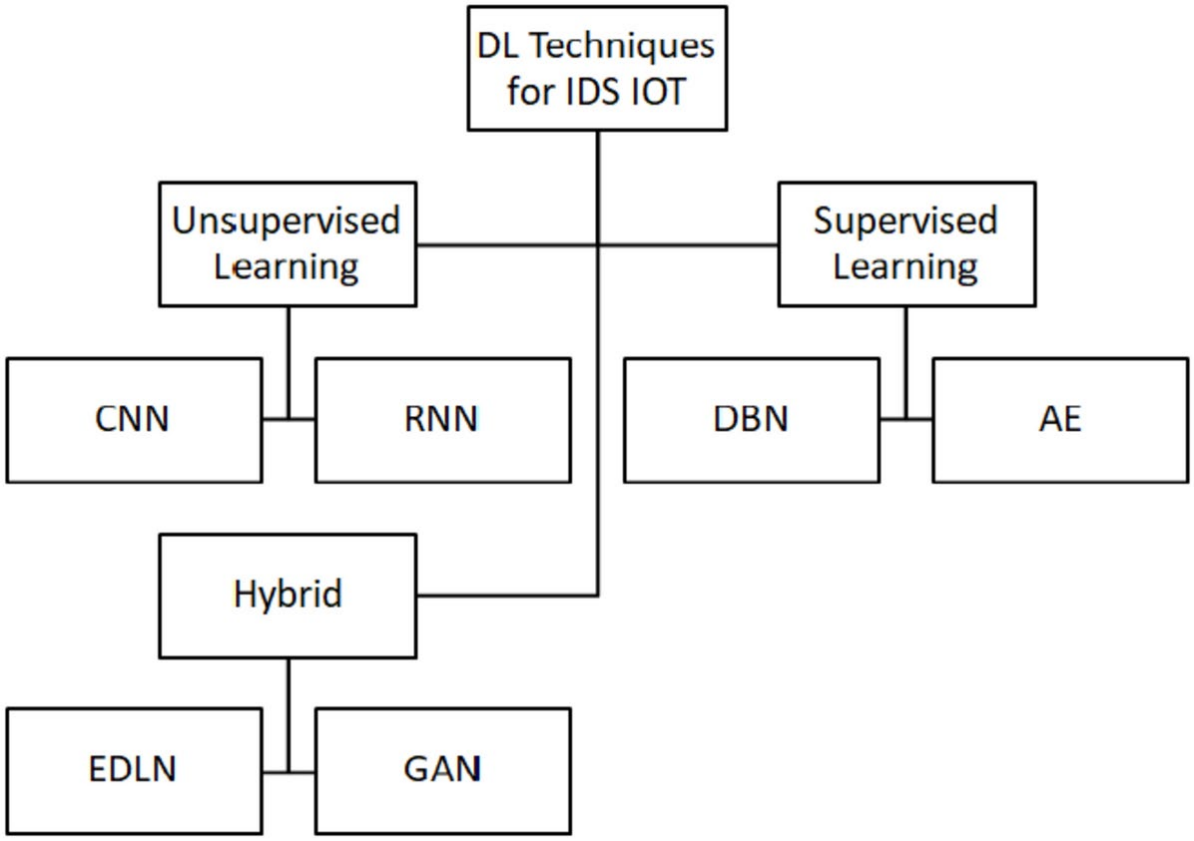
DL Techniques for IDS in IoT.

Most detection techniques are based on ML algorithms during the IDS training phase. Various ML techniques are presented. Table 1 briefly overviews ML methods used in previous related work and their advantages and limitations. Table 2 summarizes the research on IDSs using different datasets and ML/DL techniques. Last, IDSs in IoT networks are designed using standard ML techniques, as shown in Figs. 2 and 3^17^.

**Table 1:** Various machine learning methods for IoT systems.

ML Method	Attacks	Pros	Cons
KNN^18,19^	U2R, R2L, Flooding attacks, DoS, DDoS	Simplicity	There are two challenges: one is to find the best value of K and identify missing nodes
DT^19,20^	DDoS^19^, U2R, R2L^20^	Easy and simple to use method	It needs ample storage Ease to use if DTs are few, but it’s always complex in computation
SVM^21–23^	Scan, DDoS (TCP, UDP flood), smurf, port sweep	Data with several features SVMs use less storage and memory	It isn’t easy to understand and interpret

**Table 2:** Some studies that used machine and deep learning techniques in IoT Security.

Study	ML			DL			Dataset	Thread
	KNN	EL	RF	CNN	DBN	AE		
^24^	–	✓	–	–	–	–	KDD99	Network Traffic anomaly detection
^25^	–	–	–	–	–	✓	Outlier Detection Datasets	Anomaly detection
^26^	–	–	–	✓	–	–	500 samples for the dataset	Anomaly detection
^27^	✓	–	–	–	–	–	NSL-KDD	DoS, Probe, R2L, U2R
^28^	–	–	✓	–	–	–	KDD CUP 99	DoS, Probe, R2L, and U2R

Figure 2 shows ML algorithms divided into Supervised and Unsupervised ML^23^. To solve classification and regression tasks in machine learning, one of the best Supervised learning techniques is Support Vector Machine (SVM)^29^; SVMs are good at resolving binary classification issues, which require dividing the data set’s elements into two categories. Support vector machine methods find the best decision line, which creates two groups of data points. While working with high-dimensional feature spaces, this boundary is called a hyperplane.

Because SVMs are based on statistical learning, they are ideal for anomaly detection when it’s essential to categorize data into regular and unusual classes^23^. Methods such as anomaly-based intrusion detection and online learning in real-time are applied by SVMs Because of their simplicity, making them highly scalable^21–23^. In^30^, authors have used an optimized version of SVM related to the Internet of Vehicles (IoV) networks. No parameters are needed when using K-Nearest Neighbors (KNN). Euclidean measures the distance between K-Nearest Neighbors (KNN) without any parameters. Distance between neighbors is calculated by Euclidean distance^30^. Several studies^18,31–33^ use KNN-based classification as a general method in anomaly intrusion detection. KNN is simple and accurate, but finding missing nodes and determining the optimal value for k takes time and money. Decision Tree DT was used in a study published in^19^ to identify malicious sources in network traffic and detect DDoS attacks in an IoT environment. According to certain studies^34,35^, RF is suitable for detecting anomalies and intrusions in IoT networks.

#### DL techniques for IDS

To establish a satisfying comparison with other earlier work, we will emphasize NN, similar to our model, in the previous Table that compares some ML and DL methodologies. Regarding huge dataset applications, DL algorithms perform better than ML techniques. Due to the massive and diverse volumes of data produced in IoT contexts, DL becomes especially pertinent in IoT security applications^36^. Moreover, deep learning can automatically create intricate feature sets using sample data^36^. Deep linking in the Internet of Things networks is made possible by DL algorithms, which is another benefit^37^; it makes it possible for IoT-based systems to auto-connect with one another to complete predetermined collaboration tasks without the need for human participation^28^.

DL can extract structured representations of features in complex deep architecture; that’s why it is a subset of ML techniques that extract feature sets using several non-linear processing layers. Following any required changes, these feature sets are used to recognize abstract patterns^38^. Figure 3 shows three DL applications: discriminative with supervised learning, generative with unsupervised learning, and a hybrid combining of the two modes.

## Proposed model

**Fig. 4:**
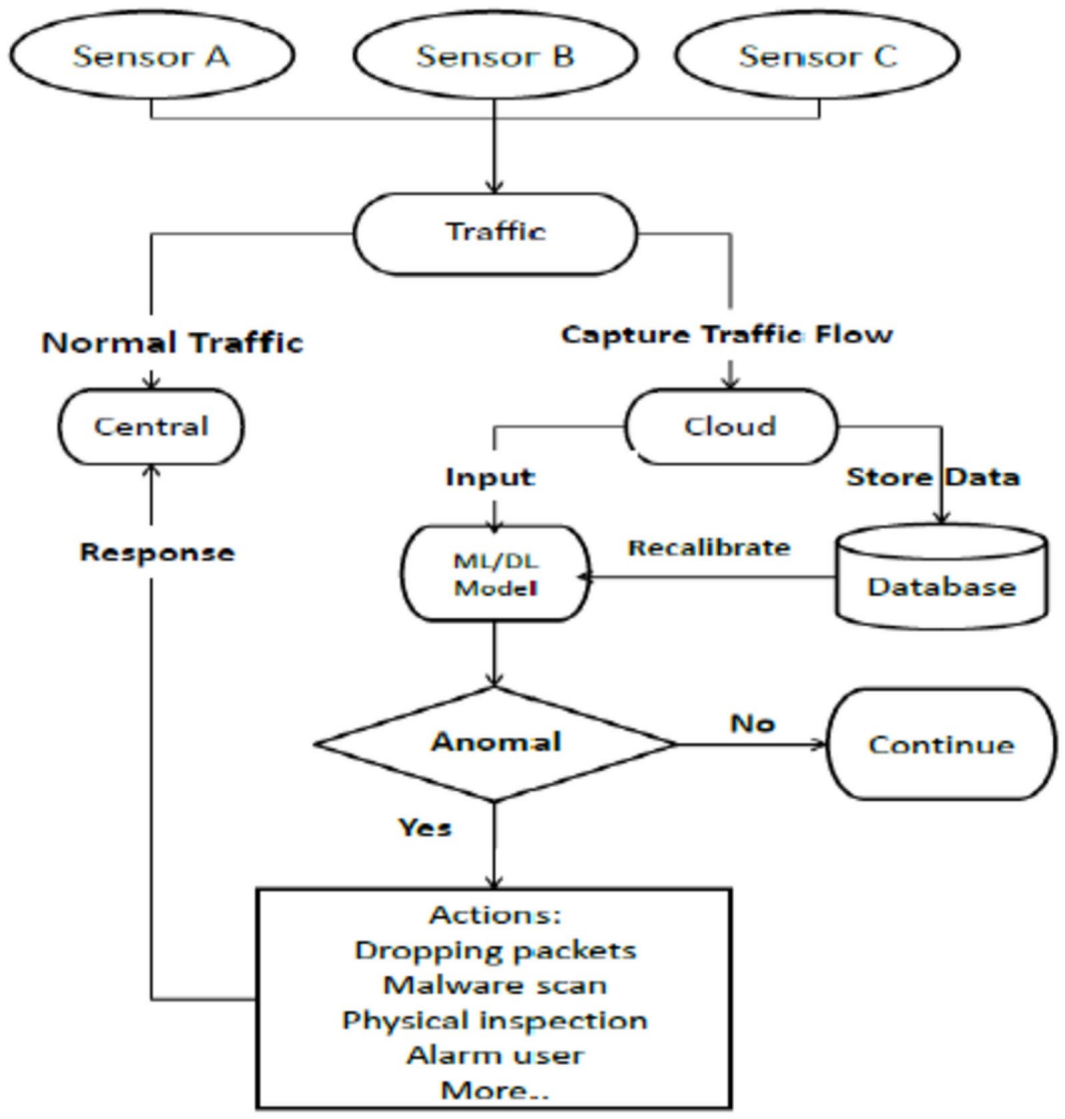
The proposed anomaly detection system model.

As shown in Fig. 1, this work proposes an anomaly detection model for IoT security, while another target is using machine and deep learning methods to identify the best design choice. Figure 4 shows. The first phase in the process is done by the traffic capture unit, which records traffic flow from sensors to the central unit. A computing unit can be a local or cloud computer used to gather data on traffic flow. A Computing unit can run different machine and deep learning models and then separately calculate each model’s costs and performance, and it can save traffic flow for upcoming research or model recalibration. After testing various models, the user will choose the optimal performance model for anomaly detection.

Communication between the central and the computing unit is done by sending messages or commands, such as deleting packets, searching for malware, performing a physical inspection, giving marks to IP addresses, and alerting users when it detects anomalies. If we consider how the user would select the ML/DL model, we can assume they will do it based on performance metrics like accuracy and time savings. It’s essential to provide the optimal solution for each unique user, although an anomaly IDS for IoT security is not applied in all their situations. Our suggested approach records traffic flow and stores it in a database to enhance performance; later, the new dataset can be used for recalibration.

Algorithms are trained on data. Then, to identify any anomalies in the system, ding computation on the local machine or in the cloud. Depending on the algorithm taught, we can conclude from the results by dividing the dataset into two groups: trained and tested. When an anomaly is discovered, and based on the outcome, action is necessary, like dropping packets, alerting users, putting the sender’s IP address in the blocklist, and more. Next, the system can be physically inspected and scanned for malware.

### Dataset

#### Datasets available for IoT security & used datasets

A reliable, up-to-date dataset with both benign and abnormal activity should be assessed to evaluate the efficacy of IDS. Due to the lack of other datasets for around 20 years, most early IDS research was predicated on the KDD99^39^ dataset. Nevertheless, research indicates that the dataset of KDD99 negatively affects the IDS outcomes in^40–42^. Several investigations have been conducted to tackle the shortcomings of KDD99 and subsequent datasets. Here is a summary of the most used datasets for IDS evaluation. If we compare Synthetic & Real datasets, we will find cons and pros in both. Still, the benefits of using real datasets are more significant than synthetic data because actual data experiments are applicable in real life. Let’s talk about IoT23 as one of the Real data. In previous related work, one of their significant challenges was applying their studies and using their models on accurate data^42^ in their future work. They said applying their work to natural IoT environments is one of their challenges. We solved this challenge here. Most previous researchers discussed this, and many papers have discussed the difference between synthetic and natural datasets^43^.

#### Dataset

IoT-23 has twenty malware captures done in IoT devices, and three grabs are made for benign IoT device traffic; its size is about 50 GB, has 23 scenarios (3 benign and 20 malicious), and the number of packets Varies per scenario, ranging from thousands to millions. The dataset in its complete form contains .pcap files, which are the original network capture files, conn.log.labeled files, created by running the Zeek network analyzer, include details and information about each capture.

**Fig. 5:**
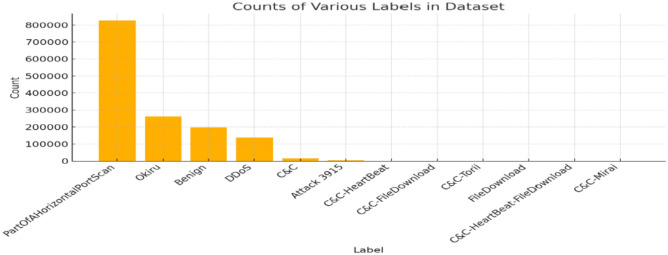
Label counts attack types.

This is because it is easier to work exclusively with the conn.log.labeled files. The data set contains 325,307,990 captures, of which 294,449,255 are malicious. The .pcap files were analyzed manually to identify the properties of the different labels. Then, a Python script was run through the log files to add labels based on the analysis. The malware file sizes varied from a few kilobytes to about 10 gigabytes. The unit of study is a flow. Although the IoT-23 dataset is multi-labelled, the labels have similar classes. Labels are the different types of attacks. For example, a label could be C&C or PartOfAHorizontal PortScan, and both have different meanings, whereas a class could be C&C-PartOfAHorizontalPortScan, which means that both malware attacks are present for the flows in this class^44^. The labels are described below, and Table 3 and Fig. 5 also show the malware labels for the IoT-23 dataset.

**Table 3:** Shows the type of attacks and its count.

Label	Count
PartOfAHorizontalPortScan	825,939
Okiru	262,690
Benign	197,809
DDoS	138,777
C&C	15,100
Attack 3915	3915
C&C-HeartBeat	349
C&C-FileDownload	43
C&C-Torii	30
FileDownload	13
C&C-HeartBeat-FileDownload	8
C&C-Mirai	1

- *Attack* A type of attack from the infected device to another host where it tries to take advantage of a vulnerability.
- *Benign* No suspicious or malicious activities were found in the connections.
- *C&C* The infected device was connected to a CC server.
- *DDoS* A Distributed Denial of Service attack is being executed by the infected device.
- *FileDownload* A file is being downloaded to our infected device.
- *HeartBeat* The packets sent on this connection are used by the C&C server to track the infected host.
- *Mirai* The connections have characteristics of a Mirai botnet.
- *Okiru* The connections have characteristics of an Okiru botnet.
- *PartOfAHorizontalPortScan* The connections scan horizontal ports to gather information for further attacks.
- *Torii* The connections have characteristics of a Torii botnet.

#### Data preprocessing

RESULT OF PRE_PROSSESING: (IoT23 combinedN.csv file). The IoT23 combinedN.csv file contains a total of 1,444,674 records. This dataset has a feature set described in Table 4 below, and we manually select the feature set to avoid loss of essential features and redundancy. Moreover, the combined file, as shown in Table 3, has 10 types of attack, including Part of a Horizontal Port Scan, Okiru, DDoS, Attack, C&C-HeartBeat, C&C File Download, C&C-Torii, File Download, C&C-Heart Beat-File Download, and C&C-Mirai. The combined dataset is divided into a training dataset size of 0.8 and a testing dataset with a length of 0.2.

**Table 4:** Description of selected IoT-23 Features.

	Feature	Description
1	duration	Duration of the network connection (in seconds)
2	orig_bytes	Number of bytes sent by the originator (source)
3	resp_bytes	Number of bytes sent by the responder (destination)
4	missed_bytes	Number of bytes missed due to dropped packets or data loss
5	orig_pkts	Total number of packets sent by the originator (source)
6	orig_ip_bytes	Total number of bytes, including headers, sent by the originator
7	resp_pkts	Total number of packets sent by the responder (destination)
8	resp_ip_bytes	Total number of bytes, including headers, sent by the responder
9	proto_icmp	Indicates if the connection uses the ICMP protocol (1 if true, 0 if false)
10	proto_tcp	Indicates if the connection uses the TCP protocol (1 if true, 0 if false)
11	proto_udp	Indicates if the connection uses the UDP protocol (1 if true, 0 if false)
12	conn_state_OTH	Connection in an “other” state (unusual states not categorized)
13	conn_state_REJ	The responder rejected the connection
14	conn_state_RSTO	The originator reset the connection
15	conn_state_RSTOS0	The originator sent a reset, and no response was received
16	conn_state_RSTR	The responder reset the connection
17	conn_state_RSTRH	The responder reset the connection after a handshake
18	conn_state_S0	A connection attempt was seen, but no reply was received
19	conn_state_S1	A connection was established, but no data was transferred
20	conn_state_S2	The connection was established, and the originator sent data but received no response
21	conn_state_S3	The connection was established, and the responder sent data but received no originator response
22	conn_state_SF	Connection fully established and terminated normally
23	conn_state_SH	A connection attempt was seen with a SYN-ACK, but there was no response from the originator
24	conn_state_SHR	A connection attempt was seen with a SYN-ACK, followed by a reset

### Performance evaluation and analysis

**Fig. 6:**
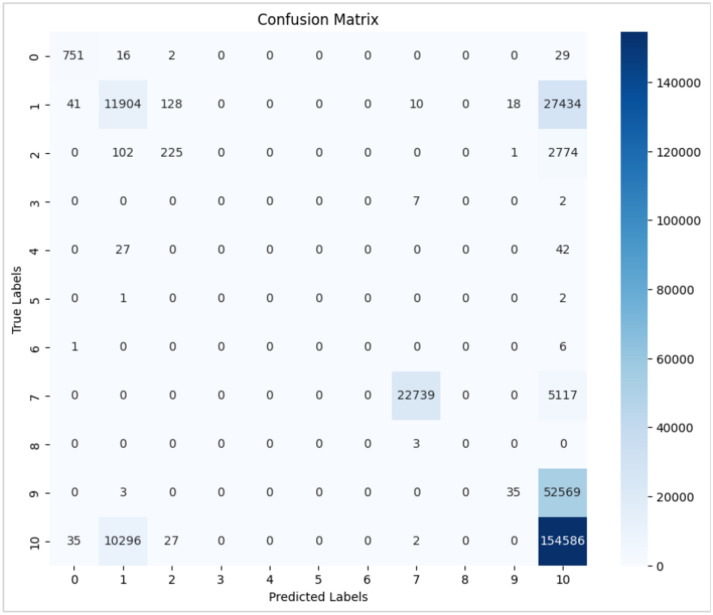
Confusion matrix.

This section will discuss the results, showing each algorithm’s confusion matrix to calculate the anomaly. Figure 6 shows the confusion matrix, which summarizes the model’s performance. The matrix is then visualized as a heatmap for more straightforward interpretation.

#### Hardware used and environment settings

We use a PC with AMD E1-6010 APU, 1350 MHz processor, two cores, two logical processors, twelve GB of RAM, and AMD Radeon R2 graphics. In addition, Windows 10, Colab Notebook connected to Python 3 Google Compute Engine backend (GPU RAM:1.23/12.76 GB, DISK 27.0 /78.19 GB).

#### Evaluation of metrics

1. *True Positives (TP)* The model predicts the positive class correctly.
2. *False Positives (FP)* The model mis-predicts the positive class.
3. *Precision* Precision is a measure of calculating the correctly identified positives in a model and is given.by:
  1 $$Percision = \frac{TP}{{TP + FP}}$$
4. *False Negative (FN)* Number of actual negatives identified as positives.
5. *True Negative (TN)* Number of actual negatives that were correctly identified.
6. *Recall* It is a measure of actual number of positives that are correctly identified and is given by:
  2 $${\varvec{Recall}} = \frac{TP}{{TP + FN}}$$
7. *F1 score* Is a metric that calculates the harmonic mean of precision and recall and is considered a better measure. It is given by:
  3 $${\varvec{F}}1 = \frac{2 \times \left( 1 \right) \times \left( 2 \right)}{{\left( 1 \right) + \left( 2 \right) }}$$
8. * Support score* The support score is a measuring metric of the Python library sci-kit-learn, which indicates the number of occurrences of each label where it is true.

### Test results

#### Convolutional neural networks

**Fig. 7:**
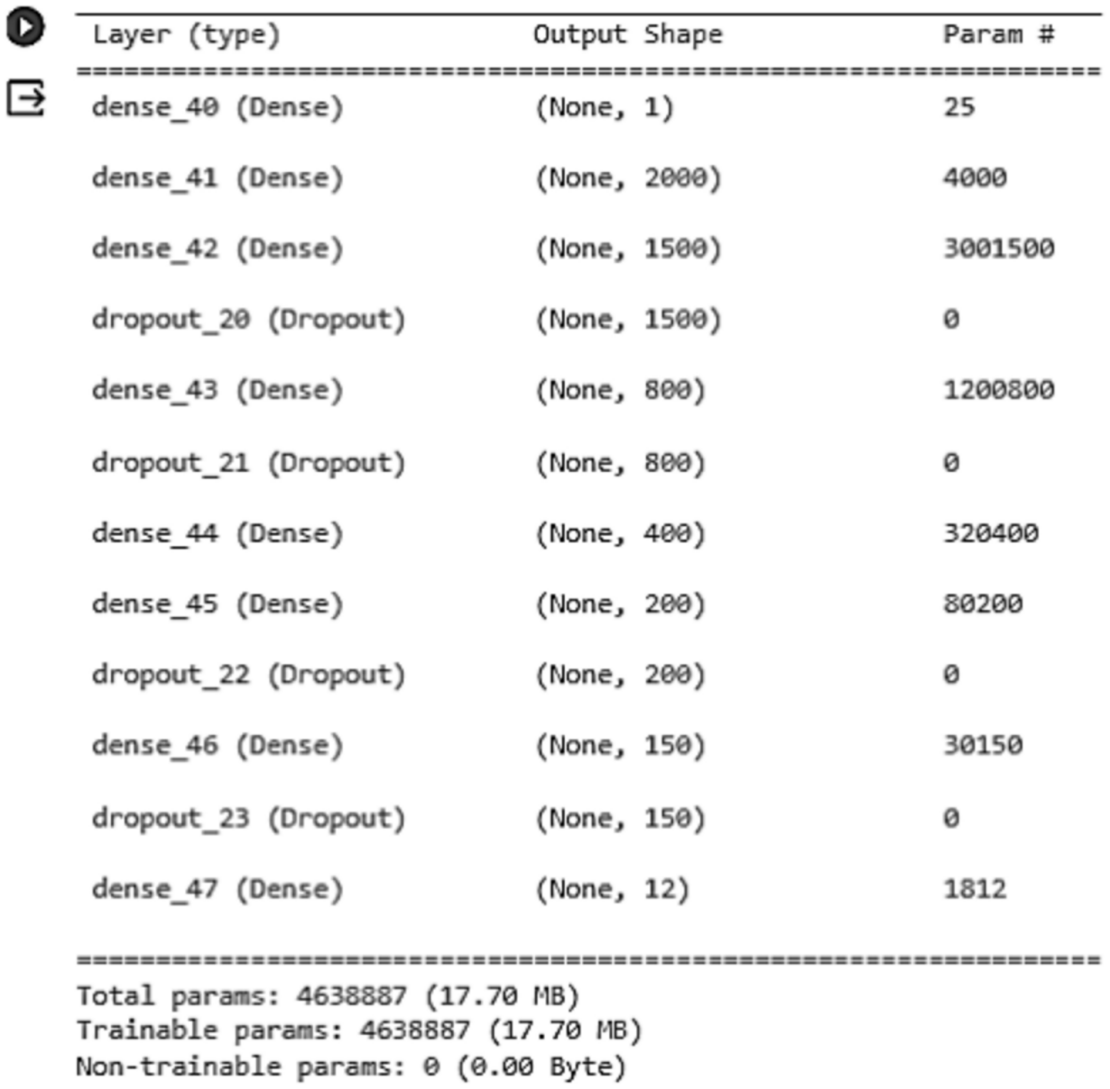
Showing our model code CNN Layers.

Deep learning has a subclass called convolutional neural networks (CNN), which require little preparation and are like the structure of neurons in the human brain. Convolutional, pooling, fully connected, and normalization are layers in CNN, which have several attributes named hyperparameters, such as input and output channels, padding size, kernels with particular width and height, etc. The neuron cluster’s output of the previous layer is combined in one neuron in the next layer in the pooling phase, which reduces the data dimension. In the fully connected layer, every neuron receives input from all the neurons in the layer before it; in contrast to the convolutional layer, individual neurons get input from one other. Figure 7 shows our model layers.

CNNs are suitable for intrusion detection in the IoT environment because they can capture unique patterns in multidimensional data. Unlike Recurrent Neural Networks (RNN) or long-short-term memory (LSTM)^45^, which focus on sequential and temporal dependencies, CNNs leverage convolutional layers that automatically learn and extract relevant features from raw data.

**Fig. 8:**
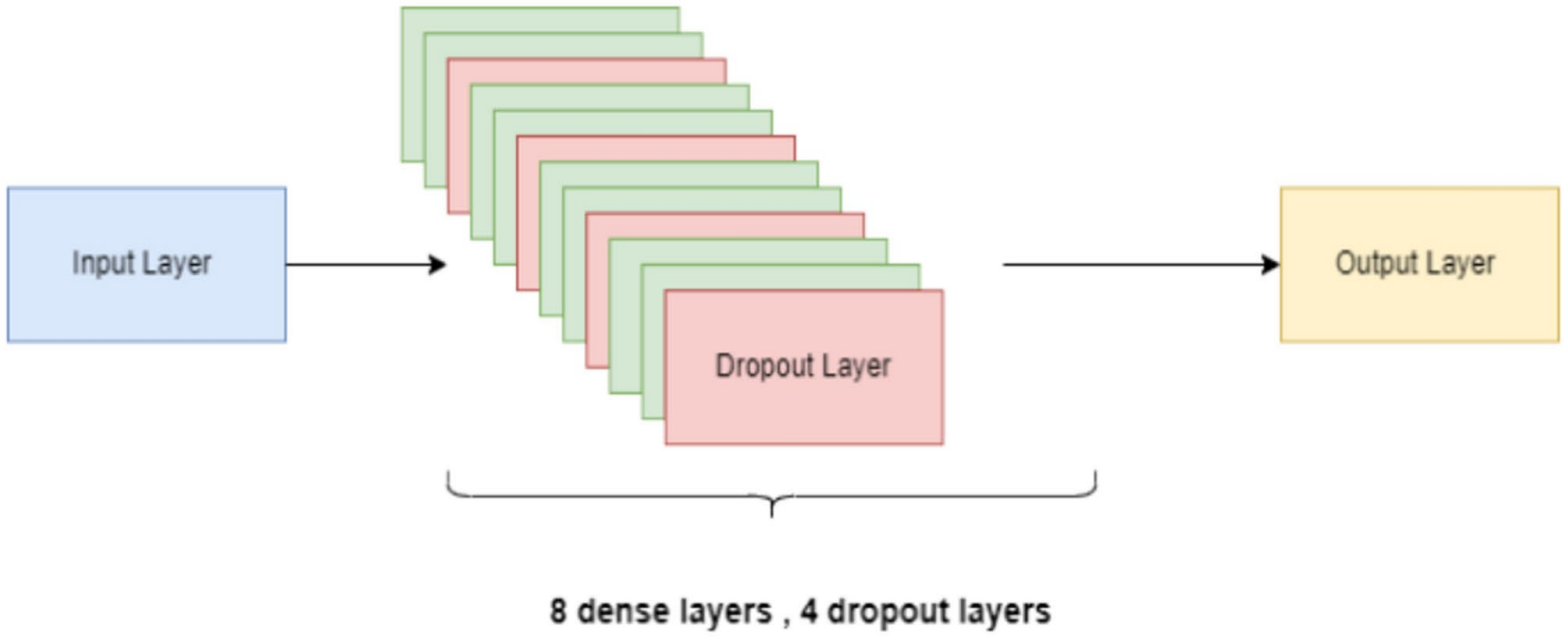
CNN model layers.

Figure 8 shows that our proposed CNN model has one input layer, eight dense layers, four dropout layers, and one output layer. The Relu is used as an activation function. If the outcome is positive, this linear function will output the input directly; if the result is unfavorable, it will output zero. The output layer’s activation function is softmax, a logistic function that normalizes the output into a probability distribution. This model uses MSE as an optimizer, a gradient descent searching algorithm. Our proposed CNN model has 4,638,887 parameters; all are trainable.

The testing accuracy of the (CNN) model in our work is 72.31%, As shown in Table 5. Our model demonstrates a significant improvement compared to the results in^46^, where the testing accuracy was reported to be 69%. Additionally, the training and testing losses in our model are considerably lower. Specifically, our testing loss is 0.0421, which is markedly better than the 0.85 reported in^43^. This substantial reduction in loss indicates that our model has a better generalization capability.

**Table 5:** CNN RESULTS.

Training accuracy	Training loss	Testing accuracy	Testing loss
0.7053	0.0327	0.7063	0.0326
0.7229	0.0300	0.7231	0.0421

We conducted experiments with two different model configurations. The results in the first row reflect the performance before adjusting the learning rate and implementing additional modifications to the model. After making these changes, as shown in the second row, the accuracy of training and testing improved while the loss decreased. These enhancements highlight that CNNs can perform better when dealing with complex datasets, particularly after fine-tuning hyperparameters and optimizing the model architecture.

As shown in Table 6, the results of the two last training models of CNN before editing learning rate and after that, and Table 7 showing CNN results of paper^46^ that we used in comparison with our model:

**Table 6:** CNN performance.

Metric	Before adjustments	After adjustments	Reference^46^
Training Accuracy	0.7053	0.7229	–
Training Loss	0.0327	0.0300	–
Testing Accuracy	0.7063	0.7231	0.6900
Testing Loss	0.0326	0.0421	0.8500

**Table 7:** CNN results of paper^46^.

Training accuracy	Training loss	Testing accuracy	Testing loss
0.6937	0.8583	0.6935	0.8602

*Accuracy improvement* Our CNN model achieved a testing accuracy of 72.31%, an improvement from the 69% reported in^46^.

**Fig. 9:**
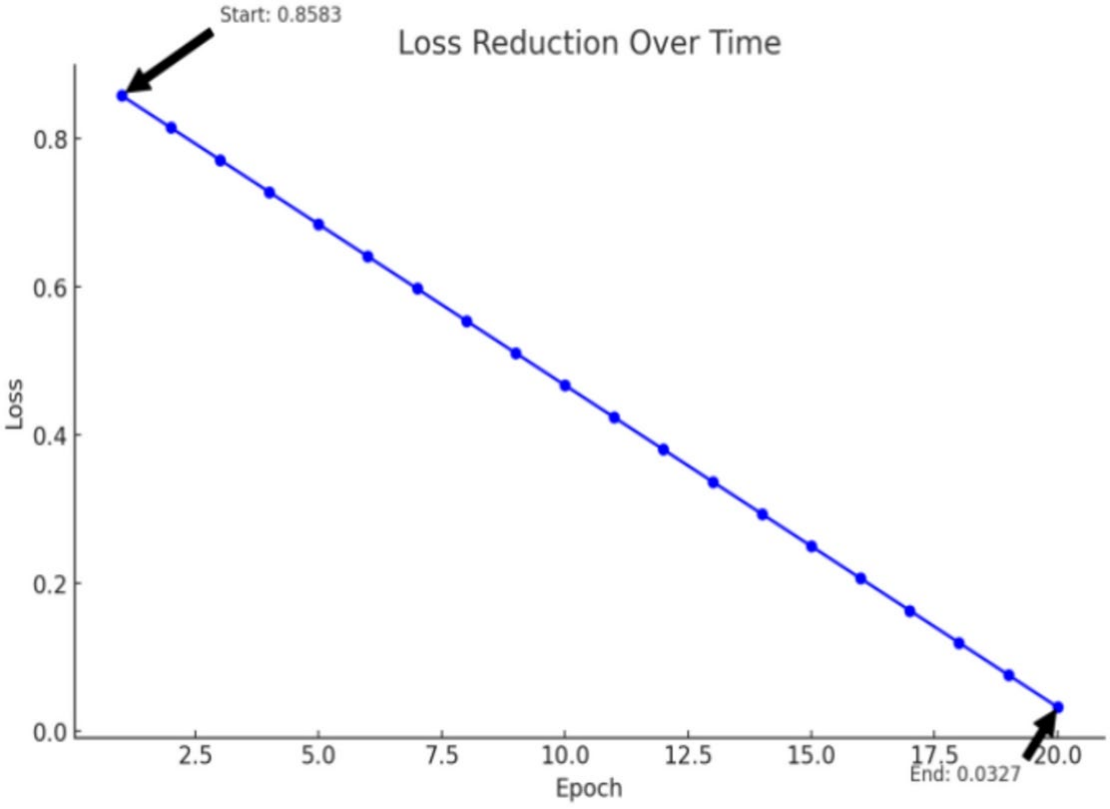
Training loss reduction over epochs.

*Loss comparison* Our model’s testing loss is significantly lower (0.0421) than the 0.85 reported in^46^, which indicates a more accurate and reliable model, as shown in Table 7 and Fig. 9.

*Model adjustments* Modifying the learning rate and making architectural changes improved the model’s performance, as reflected in the metrics before and after adjustments.

#### Results comparison

Table 8 compares testing accuracies for machine learning and deep learning methods on the IoT-23 Dataset. This comparison improves the efficiency of different approaches and observes how well they work, as shown in Fig. 11.

**Table 8:** Comparison of different ML/DL methods on the IoT-23 Dataset.

Method	Testing accuracy	Dataset used
Naive Bayes^47^	0.23	IoT-23 dataset
Naive Bayes^43^	0.30	IoT-23 dataset
CNN^47^	0.67	IoT-23 dataset
CNN^46^	0.69	IoT-23 dataset
CNN (ours)	0.72	IoT-23 dataset

The Naive Bayes method, as reported in papers^47^ and^43^, shows testing accuracies of 0.23 and 0.30, respectively. The modest increase in accuracy from paper^47^ to paper^43^ suggests some improvements in the implementation or parameter tuning. However, the overall performance remains relatively low, indicating that Naive Bayes may not be well-suited for this dataset. The inherent assumptions of Naive Bayes, such as feature independence, might not hold for the IoT-23 Dataset, leading to suboptimal performance.

**Fig. 10:**
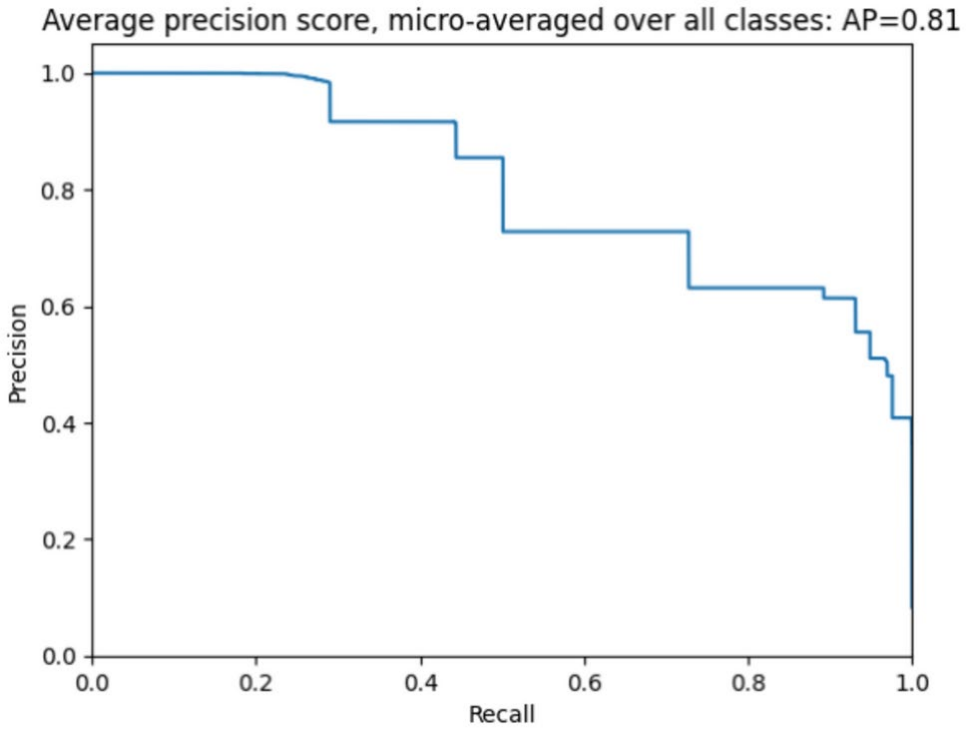
Precision and Recall for all classes.

When selecting which metric to use, we should consider other parameters, such as whether this model uses multi-class, the dataset and its number of features, and which algorithm is used. Sometimes, some metrics do not fit with ML algorithms, so this table compares with our model, which uses multiple classes and features more significantly than others. Some use only binary classes or one class. Figure 10 shows each chart for each 12-class precision and Recall. Compute precision, such as using one class of DDOS attack or selecting two classes only, is a challenge in other work we solved here. We demonstrate this by computing precision for all classes. Figure 10 shows precision and recall, and Table 9 will compare different metrics used to select which metric is suitable to improve model performance, comparing our model with other studies. In^43^, the metric used is precision, which gives 0.995 using the RF algorithm, while^46^, the best algorithm that shows high performance is DT, which offers 73% accuracy. On the other hand^48^, using only 2 classes for the NSL-KDD dataset can achieve precision = 1^49^. The recall using the CNN algorithm is 99 using multiclass for the CIDDS-001 dataset. Finally, our model suitable metric precision equal to 1 using multiclass classification improves performance. Moustafa and Slay^45^ showing different comparisons as mentioned here.

**Table 9:** Comparison of precision, Recall, and Accuracy for different datasets.

Study	METRIC	DATASET USED	MULTICLASS	BEST AI algorithm
^43^	Precision = 0.995	IoT-23	-	RF
^46^	Accuracy = 0.73	IoT-23	-	DT
^48^	Precision = 1	NSL-KDD	2-CLASS	CNN
^49^	Recall = 0.99	CIDDS-001	✓	CNN
Our	Precision = 1	IOT23	✓	CNN

**Fig. 11:**
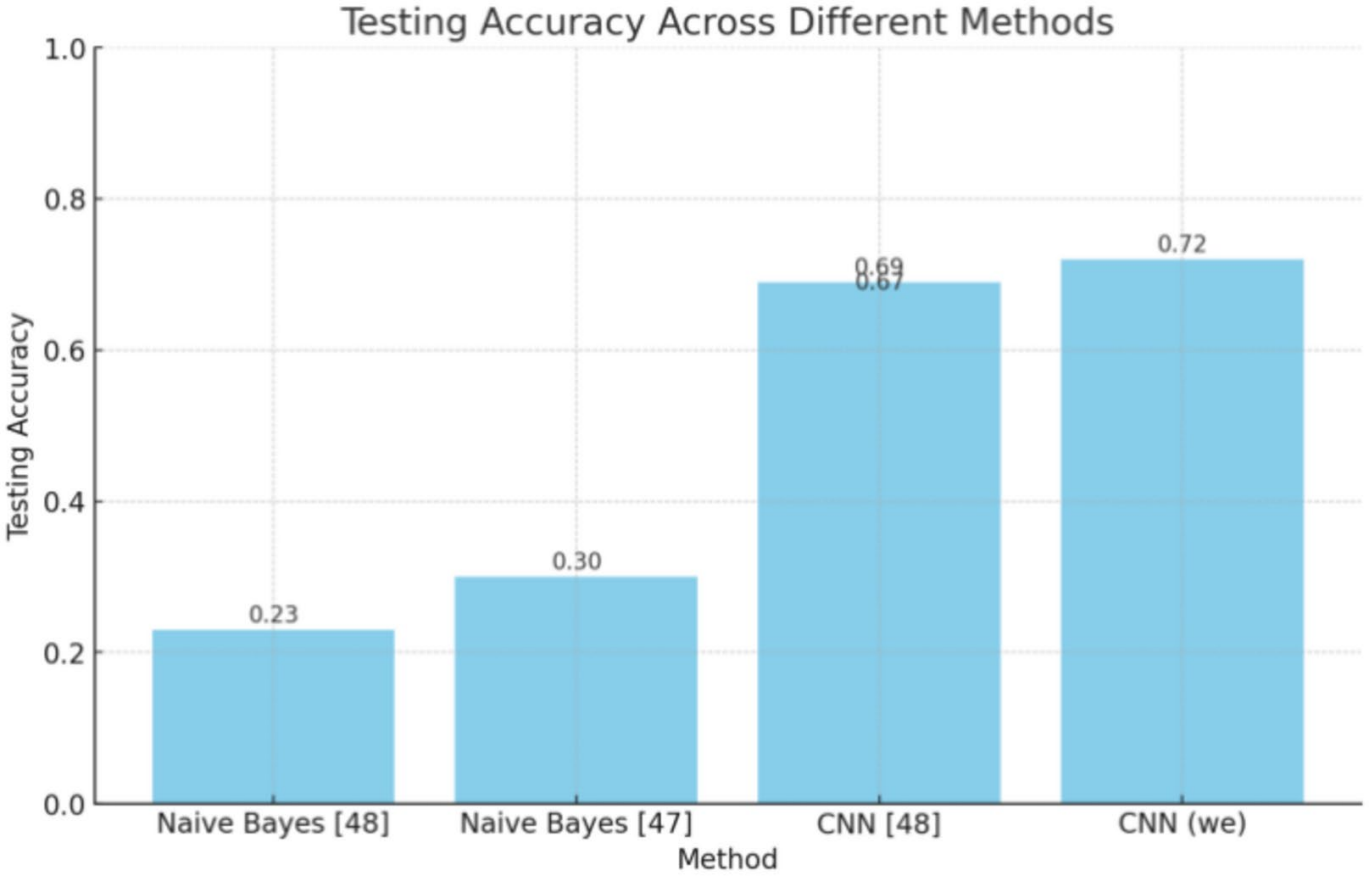
Testing accuracy across different methods.

Convolutional Neural Networks (CNNs) demonstrate substantially higher testing accuracies. The accuracy of CNN models presented in the publication^47^ is 0.67 and 0.69, a notable improvement over Naive Bayes. That highlights the strength of CNNs in capturing complex patterns and dependencies in the data, likely present in the IoT-23 Dataset. The “CNN (ours)” model has the highest testing accuracy, 0.72. This result indicates that more refinements and optimizations have been successfully applied, making it the most effective approach among those compared, as shown in comparison of Fig. 11. Several potential advancements in our CNN model include better architecture design, advanced training techniques, or enhanced data preprocessing steps. The comparison shows a clear performance gap between Naive Bayes and CNN methods, with CNNs significantly outperforming Naive Bayes for the IOT-23 Dataset, which reinforces the notion that more complex and expressive models like CNNs are better equipped to handle the intricacies of IoT data. Furthermore, the little gains in CNN performance from 0.67 to 0.72 indicate continuous developments and enhancements in this field. These improvements are crucial as they contribute to more accurate and reliable IoT systems, which can have significant real-world implications.

Table 10 strongly advocates using CNNs rather than Naive Bayes for jobs utilizing the IoT-23 Dataset. Our CNN model’s superior performance indicates the possibility of additional research and development in this field to achieve even greater accuracy and more robust models. This discussion underscores the importance of continuous innovation and evaluation in machine learning, particularly for applications in IoT.

**Table 10:** Best AI algorithm FOR different datasets.

Model (metric)	Dataset used	Best AI algorithm
Accuracy	MQTT-IoT-IDS2020	RF^50^
Accuracy	500 samples for the dataset	CNN^26^
–	CIC-IDS2017 dataset & CSE-CIC-IDS2018 dataset	CNN^51^
Precision	Bot-IoT	Naive Bayes^52^

In the context of intrusion detection systems (IDS) and anomaly detection, the table lists the top AI algorithms for different datasets and assessment metrics. Optimal AI Algorithm for MQTT-IoT-IDS2020 Dataset accuracy^50^ Random Forest (RF) for the MQTT-IoT-IDS2020 dataset. The Random Forest technique is found to be the most accurate model. Random Forest, an ensemble method, is known for its robustness and ability to handle many features effectively, making it suitable for complex datasets like MQTT-IoT-IDS2020. The Best AI Algorithm, Convolutional Neural Network (CNN), achieved high accuracy on a Dataset of 500 Samples^26^. For a smaller dataset with 500 samples, CNN is highlighted as the top performer. Convolutional neural networks (CNNs) are also the best AI algorithms for overall performance on the CIC-IDS2017 and CSE-CIC-IDS2018 datasets^51^. CNN is recognized as the best-performing algorithm for the combined CIC-IDS2017 and CSE-CIC-IDS2018 datasets. These datasets are comprehensive and include a variety of attacks, highlighting CNN’s capacity to generalize well across diverse types of intrusion data. The choice of CNN underscores its superior performance in capturing intricate patterns associated with different intrusions. Precision on Bot-IoT Dataset, the Best AI Algorithm is Naive Bayes^52^.

The Table provides insights into the suitability of different AI algorithms for various datasets and evaluation metrics. Key observations include CNN’s versatility: CNNs appear effective across multiple datasets and metrics, indicating their strong ability to model complex data patterns.

## Conclusion and future works

Research highlights the considerable benefits of Convolutional Neural Networks (CNN) over exciting enhanced deep learning approaches and traditional machine learning.

Our thorough evaluation of benchmark datasets, including MQTT-IoT-IDS2020 and Bot-IoT, demonstrated that the proposed model delivers higher accuracy while maintaining a high precision rate, which is crucial for reducing false positives in real-world applications. Our CNN-based model successfully combats overfitting and adapts to IoT-specific threat patterns by utilizing advanced data augmentation and regularization techniques. These enhancements emphasize the potential of deep learning in strengthening IoT security frameworks. Looking ahead, this model sets a new benchmark for future IDS research and development, providing a scalable and resilient solution to protect IoT networks from increasingly complex cyber threats. Recently, IDS applications for IoT have varied in different areas, such as healthcare and the internet of drones^53,54^.

Despite the notable advancements achieved in this study, several challenges persist in using deep learning for intrusion detection in IoT. A key challenge is the constant evolution of cyber threats, necessitating models that can adapt and learn from new, previously unseen attack types. Ensuring the IDS can dynamically update and improve without extensive re-training is essential for maintaining its long-term effectiveness.

## Data Availability

Data is available upon request from the first author, Marina S. Amine (marina.saed@fci.suezuni.edu.eg).
